# Intestinal Region-Dependent Alterations of Toll-Like Receptor 4 Expression in Myenteric Neurons of Type 1 Diabetic Rats

**DOI:** 10.3390/biomedicines11010129

**Published:** 2023-01-04

**Authors:** Nikolett Bódi, Abigél Egyed-Kolumbán, Benita Onhausz, Bence Pál Barta, Afnan AL Doghmi, János Balázs, Zita Szalai, Mária Bagyánszki

**Affiliations:** Department of Physiology, Anatomy and Neuroscience, University of Szeged, 6726 Szeged, Hungary

**Keywords:** toll-like receptors, toll-like receptor 4, enteric nervous system, myenteric neurons, type 1 diabetes, hyperglycemia, insulin, gut-region specificity

## Abstract

Toll-like receptor 4 (TLR4) can activate pro-inflammatory cascades in the gastrointestinal tract. Our aim was to determine TLR4 expression in myenteric neurons of different gut regions using a type 1 diabetic model. Ten weeks after the onset of hyperglycemia, myenteric whole-mount preparations from the duodenum, ileum and colon of streptozotocin-induced diabetic, insulin-treated diabetic and control rats were prepared for TLR4/peripherin double-labelling fluorescent immunohistochemistry. Immunogold electron microscopy was applied to evaluate TLR4 expression in the myenteric perikaryon and neuropil. Tissue TLR4 levels were measured by enzyme-linked immunosorbent assay. In controls, the number and proportion of the TLR4-immunoreactive myenteric neurons showed an increasing tendency to aboral direction. These values were significantly higher in diabetics compared to controls in the duodenum and ileum, but were significantly lower in the colon. In diabetics, the distribution of TLR4-labelling gold particles between the perikaryon and neuropil of myenteric neurons varied in a different way by intestinal segment. TLR4 tissue concentration changed only in the diabetic duodenum, and it decreased in muscle/myenteric plexus-containing homogenates, while it increased in mucosa/submucosa/submucous plexus-containing samples relative to controls. Insulin had beneficial effects on TLR4 expression. These findings support that chronic hyperglycemia has segment-specific effects on TLR4 expression, contributing to gastrointestinal disorders in diabetic patients.

## 1. Introduction

Activated inflammatory pathways [1,2,3] have harmful effects on the structure and function of the enteric nervous system in diabetes [4], which in severe or longer-term cases can even lead to enteric neuropathy [5,6,7]. The details of this complex process have not yet been fully revealed, but it is clear that not only the intercellular processes of the enteric neurons, but also the microenvironment of the enteric ganglia is decisive in the development of diabetic neuropathy.

Because of the lack of blood-brain-barrier in the periphery, the enteric ganglia are in close contact with their environment [8]. The enteric ganglia are not vascularized; the enteric neurons and glia cells are supplied by the microcirculation of the gut wall. Our previous study on type 1 diabetic rats showed that the intestinal microvessels supplying myenteric ganglia may be targets of diabetic damage and contribute to diabetes-related neuropathy [5,9].

The enteric plexuses and the different types of enteric glia cells encase the entire intestinal wall, and thus come into contact with various cells, such as goblet cells, Microfold cells, lymphocytes, macrophages etc. of the intestinal wall [10]. By this neuro-immune interaction, the enteric nervous system and the resident innate and adaptive immune cells together regulate intestinal functions in health and disease. Both the nervous system and the immune system sense and react to the dynamic intestinal environment inside the gut wall and the gastrointestinal tract lumen [11]. One important factor in the intestinal milieu is the huge number of bacteria, archeae, fungi and protists, these being the microbes which constitute the microbiome of the gut [12,13]. The effect of metabolic diseases on the composition of the intestinal microbiome has been extensively studied during the past decade [14,15,16,17]. These results show that intestinal dysbiosis, the imbalance in the microbial gut community, can significantly contribute to the development of both type 1 and type 2 diabetes [18,19].

Toll like receptors (TLRs) belong to the pattern recognition receptor (PRR) family and play a crucial role in innate immunity by recognizing pathogen-associated molecular patterns (PAMPs). TLRs are expressed in numerous cell populations in the body, including the immune, neural, and glial cells [20]. The microbial signals from the intestinal lumen can stimulate TLRs and activate different downstream cascades. TLR4 can sense microbial lipopolysaccharides (LPS), which are cell components of Gram-negative bacteria, and their amount was found to be elevated in metabolic diseases, such as insulin resistance or obesity [21]. LPS via TLR4 can activate pro-inflammatory pathways in the gastrointestinal tract [16,22], thus TLRs may play a crucial role in diabetic enteropathy. Therefore, our aim was to determine the proportion of TLR4-immunoreactive (IR) myenteric neurons and TLR4 subcellular localization along the duodenum-colon axis, and, furthermore, to evaluate the expression of TLR4 in different intestinal layers and gut regions using a type 1 diabetic rat model.

## 2. Materials and Methods

### 2.1. Animal Model

Adult male Wistar rats (Toxi-Coop Zrt., Balatonfüred, Hungary) weighing 200–300 g, kept on standard laboratory chow (Farmer-Mix Kft., Zsámbék, Hungary), and with free access to drinking water, were used throughout the experiments. The rats were divided randomly into three groups: diabetics (n = 12), insulin-treated diabetics (n = 12) and sex- and age-matched controls (n = 15). Hyperglycemia was induced by a single intraperitoneal injection of streptozotocin (STZ, Sigma-Aldrich, Budapest, Hungary) at 60 mg/kg [5,6,23]. The animals were considered diabetic if the non-fasting blood glucose concentration was higher than 18 mmol/L. From this time on, the insulin-treated group of hyperglycemic rats received a subcutaneous injection of insulin (Humulin M3, Eli Lilly Nederland, Utrecht, The Netherlands) each morning (3 IU) and afternoon (2 IU). Equivalent volumes of saline were given subcutaneously to the diabetic and the control rats. The blood glucose level and weight of each animal were measured weekly. Those diabetic animals that recovered spontaneously, or whose glucose level decreased under 18 mmol/L during the 10-week experimental period, were excluded from the study. In all procedures involving experimental animals, the principles of the National Institutes of Health (Bethesda, MD, USA) guidelines and the EU directive 2010/63/EU for the protection of animals used for scientific purposes were strictly followed, and all of the experiments were approved by the National Scientific Ethical Committee on Animal Experimentation (National Competent Authority), with the license number XX./1636/2019.

### 2.2. Tissue Handling

Ten weeks after the onset of hyperglycemia, the animals were sacrificed by cervical dislocation under chloral hydrate anaesthesia (375 mg/kg i.p.). The gut segments of diabetic, insulin-treated diabetic and control rats were dissected and rinsed in a 0.05 M phosphate buffer (PB; pH 7.4). Samples were taken from the duodenum (1 cm distal to the pylorus), the ileum (1 cm proximal to the ileo-cecal junction) and the proximal colon, and processed for fluorescent immunohistochemistry, quantitative electron microscopy and enzyme-linked immunosorbent assays (ELISA). For double-labelling fluorescent immunohistochemistry, the intestinal segments were cut along the mesentery, pinched flat, and fixed overnight at 4 °C in 4% paraformaldehyde solution buffered with 0.1 M PB (pH 7.4). The samples were then washed, the mucosa, submucosa, and circular smooth muscle were removed, and whole-mounts with the myenteric plexus adhering to the longitudinal muscle were prepared. For paraffin sectioning, samples (2–3 mm) from different gut segments were fixed in 4% paraformaldehyde and embedded in melted paraffin. For post-embedding electron microscopy, small pieces (2–3 mm) of the gut segments were fixed in a 2% paraformaldehyde and 2% glutaraldehyde solution and then further fixed for 1 h in 1% OsO4. After rinsing in buffer and dehydrating in increasing ethanol concentrations and acetone, they were embedded in Embed812 (Electron Microscopy Sciences, Hatfield, PA, USA). For the ELISA, the 3-cm-long gut segments were cut along the mesentery and pinched flat. The layer of mucosa and submucosa containing the submucous plexus as well as the layers of intestinal smooth muscle layers including the myenteric plexus were snap-frozen in liquid nitrogen and stored at −80 °C until use.

### 2.3. Fluorescent Immunohistochemistry

For double-labelling immunohistochemistry, whole-mount preparations derived from different gut segments were immunostained with TLR4 and peripherin, while a TLR4 and S100 immunohistochemistry was performed on intestinal paraffin sections. Briefly, after blocking in tris (hydroxymethyl)aminomethane-buffered saline (TBS) containing 1% bovine serum albumin and 10% normal goat serum, the whole-mounts were incubated overnight with anti-TLR4 (mouse monoclonal IgG; SAB1404475, Sigma-Aldrich, Hungary; final dilution 1:100) and pan-neuronal anti-peripherin (rabbit polyclonal IgG; AB1530, EMD Millipore Corporation, Burlington, MA, USA; final dilution 1:400) primary antibodies at 4 °C. In the case of paraffin sections, anti-TLR4 (mouse monoclonal IgG; SAB1404475, Sigma-Aldrich, Hungary; final dilution 1:100) and glial marker anti-S100 (rabbit polyclonal IgG; Z0311, DakoCytomation, Glostrup, Denmark; final dilution 1:400) were used as primary antibodies. After washing in TBS with 0.025% Triton X-100, sections were incubated with anti-mouse CyTM3 (Jackson ImmunoResearch Laboratories, West Grove, PA, USA; final dilution 1:200) and anti-rabbit Alexa Fluor 488 (Life Technologies Corporation, Molecular Probes, Carlsbad, CA, USA; final dilution 1:200) secondary antibodies for 1 h at room temperature. Negative controls were performed by omitting the primary antibody when no immunoreactivity was observed. Whole-mounts were mounted on slides in Fluoromount^TM^ Aqueous Mounting Medium (Sigma-Aldrich, Hungary), and observed and photographed with a Zeiss Imager Z.2 fluorescent microscope (Jena, Germany) equipped with an Axiocam 506 mono camera. One hundred myenteric ganglia were taken from each intestinal segment from each experimental group, and the numbers of TLR4-IR and peripherin-IR neurons, and those myenteric neurons in which the two markers were colocalized (per ganglia), were counted. The percentage of ganglia containing TLR4-IR myenteric neurons was also determined.

### 2.4. Quantitative Post-Embedding Immunohistochemistry

Four Embed blocks originating from each intestinal segment and condition were used to prepare ultrathin (70 nm) sections, which were mounted on nickel grids and processed for TLR4 immunogold labelling. The sections (three grids per block) were incubated overnight in anti-TLR4 mouse monoclonal IgG (SAB1404475, Sigma-Aldrich, Hungary; final dilution 1:100) primary antibody, followed by colloidal gold conjugated anti-mouse IgG (conjugated to 18 nm gold particles; Jackson ImmunoResearch, USA; final dilution 1:20) secondary antibody for 3 h. The specificity of the immunoreaction was assessed in all cases by omitting the primary antibodies in the labelling protocol and incubating the sections only in the gold conjugated secondary antibodies. Sections were counterstained with uranyl acetate (Merck, Darmstadt, Germany) and lead citrate (Merck, Germany), and were examined and photographed with a JEOL JEM 1400 transmission electron microscope. The quantitative features and the subcellular distributions of TLR4 labelling gold particles were determined in myenteric ganglia. Fifty digital photographs of five myenteric ganglia per intestinal segment per condition were made at a magnification of 20,000× with the AnalySIS 3.2 program (Soft Imaging System GmbH, Münster, Germany). TLR4 density was expressed as the total number of gold particles per unit area.

### 2.5. Measurement of Tissue TLR4 Concentrations

Intestinal tissue samples, including the mucosa and submucosa with the submucous plexus (MUC-SUBMUC-SP), or the intestinal smooth muscle layers with the myenteric plexus in between (MUSCLE-MP), were frozen in liquid nitrogen, crushed into powder in a mortar, and homogenized in 500 µL homogenizing buffer (100 µL Protease Inhibitor Cocktail (Sigma-Aldrich, Hungary) in 20 mL 0.05 M PB). Tissue homogenates were centrifuged at 5000 rpm for 20 min at 4 °C. The TLR4 level of the intestinal tissue samples were determined by means of quantitative ELISA according to the manufacturer’s instructions (GA-E0089RT, GenAsia Biotech Co., Shanghai, China). Optical density was measured at 450 nm (Benchmark Microplate Reader; Bio-Rad, Budapest, Hungary). The tissue TLR4 concentrations were expressed as ng/mg protein.

### 2.6. Bradford Protein Micromethod for the Determination of Tissue Protein Content

A commercial protein assay kit was used for the determination of protein content in tissue samples. Bradford reagent was added to each sample. After mixing and following 10 min incubation, the samples were assayed spectrophotometrically at 595 nm. The protein level was expressed as mg protein/mL.

### 2.7. Statistical Analysis

A statistical analysis was performed with one-way analysis of variance (ANOVA) with a Newman–Keuls test (table) and a Kruskal-Wallis test with Dunn’s multiple comparisons test (graphs). All analyses were carried out with GraphPad Prism 6.0 (GraphPad Software, San Diego, CA, USA). A probability of *p* < 0.05 was set as the level of significance. All data were expressed as means ± SEM.

## 3. Results

### 3.1. Weight and Glycaemic Characteristics of Type 1 Diabetic Rats

The general features of the diabetic, insulin-treated diabetic and control rats were monitored during the 10-week experimental period and are summarized in Table 1. Diabetic rats were characterized by a long-lasting chronic hyperglycemia, and their average blood glucose level was 24.73 ± 1.02 mM, which was more than four times higher than that of the controls (5.80 ± 0.14 mM). Immediate insulin treatment prevented extremely high glucose concentrations; however, the values were still higher than in the control rats (11.1 ± 0.99 mM). All the animals gained weight during the 10-week experiment, but the final body weight of diabetic rats was significantly lower as compared to insulin-treated diabetic (*p* < 0.0001) and control (*p* < 0.0001) animals.

### 3.2. Intestinal Segment-Specific Presence of TLR4-Immunoreactive Myenteric Neurons in Controls

**Figure 1:**
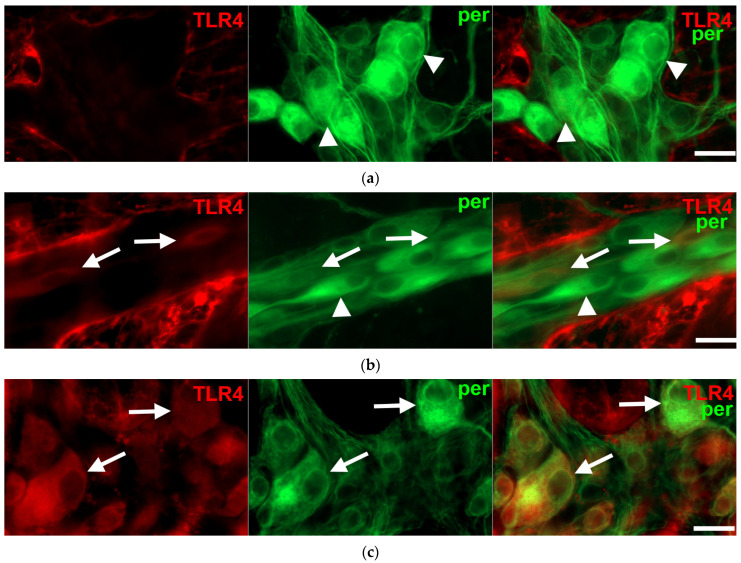
Representative fluorescent micrographs of whole-mount preparations of myenteric ganglia from the duodenum (**a**), ileum (**b**) and colon (**c**) of a control rat after TLR4-peripherin double-labelling immunohistochemistry. Peripherin was used as a pan-neuronal marker to label myenteric neurons. Arrows—TLR4-immunoreactive myenteric neurons, arrowheads—myenteric neuron, scale bars: 20 μm.

Double-labelling fluorescent immunohistochemistry revealed TLR4-IR enteric neurons on whole-mount preparations of myenteric ganglia in all investigated gut segments (Figure 1).

**Figure 2:**
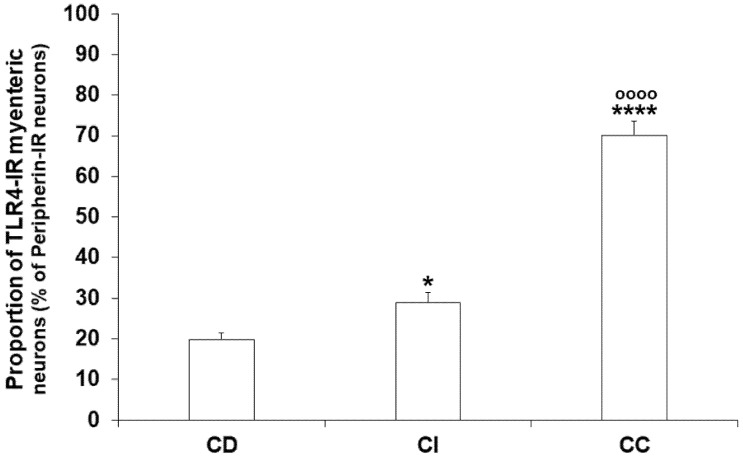
Proportion of TLR4-immunoreactive myenteric neurons of the duodenum, ileum, and colon of control rats. The proportion of TLR4-immunoreactive myenteric neurons was lowest in the duodenum, significantly higher in the ileum and highest in the colonic ganglia. Statistics: Kruskal-Wallis test with Dunn’s multiple comparisons test. Data are expressed as mean ± SEM. * *p* < 0.05, **** *p* < 0.0001 (relative to control duodenum); ^oooo^ *p* < 0.0001 (between control ileum and colon). CD—control duodenum, CI—control ileum, CC—control colon.

However, the proportion of TLR4-IR myenteric neurons displayed regional differences from segment to segment even under control conditions (Figure 2). This proportion showed an increasing tendency along the proximal-distal axis of the intestinal tract; it was lowest in the duodenum (19.74 ± 1.71%), higher in the ileum (28.99 ± 2.44%; *p* < 0.05), and highest in colonic ganglia (70.05 ± 3.43%; *p* < 0.0001) (Figure 2). On the other hand, it should be noted that not all the myenteric ganglia contained TLR4-IR cells; in the duodenum and ileum, nearly 30% of ganglia was devoid of TLR4-immunoreactivity, while in the colon only 5% were.

### 3.3. Gut Region-Dependent Induction of TLR4 Expression in Myenteric Neurons of Diabetic Rats

**Figure 3:**
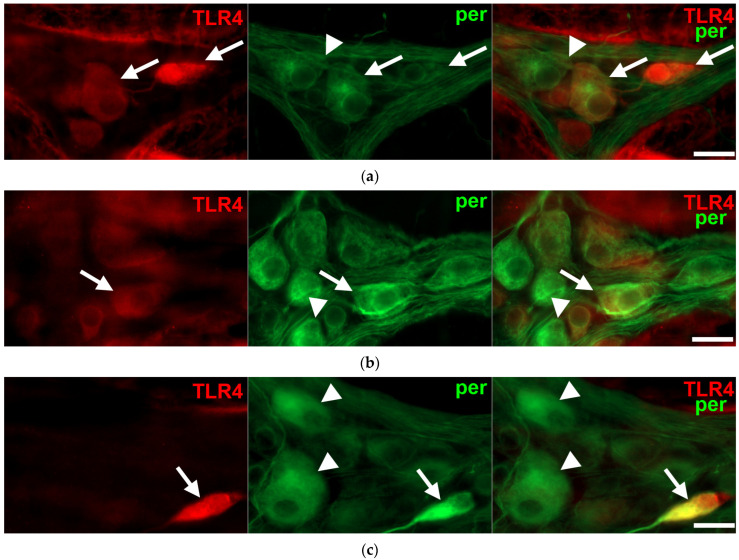
Representative fluorescent micrographs of whole-mount preparations of myenteric ganglia from the duodenum (**a**), ileum (**b**) and colon (**c**) of a diabetic rat after TLR4-peripherin double-labelling immunohistochemistry. Peripherin was used as a pan-neuronal marker to label myenteric neurons. Arrows—TLR4-immunoreactive myenteric neurons, arrowheads—myenteric neuron, scale bars: 20 μm.

**Figure 4:**
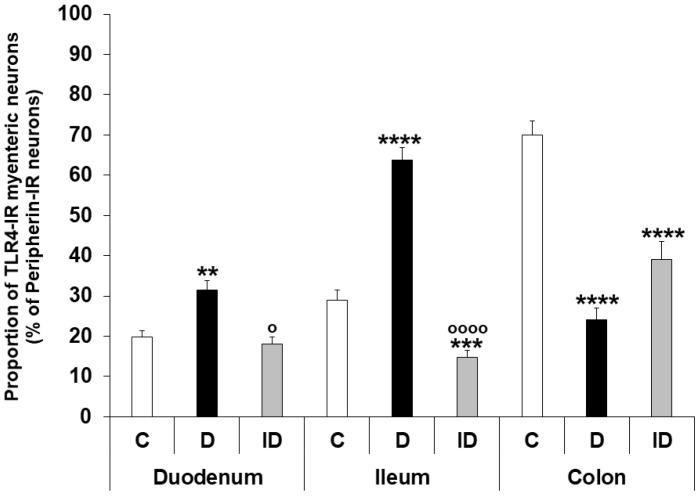
Proportion of TLR4-immunoreactive myenteric neurons of the duodenum, ileum, and colon of control, diabetic and insulin-treated diabetic rats. The proportion of TLR4-immunoreactive myenteric neurons was increased in the duodenum, robustly increased in the ileum and decreased in the colonic ganglia of diabetics relative to controls. The insulin treatment had regional protective effects on TLR4 expression. Statistics: Kruskal-Wallis test with Dunn’s multiple comparisons test. Data are expressed as mean ± SEM. ** *p* < 0.01, *** *p* < 0.001, **** *p* < 0.0001 (relative to controls); ^o^ *p* < 0.05, ^oooo^ *p* < 0.0001 (between diabetics and insulin-treated diabetics). C—controls, D—diabetics, ID—insulin-treated diabetics.

Regionally distinct changes of neuronal TLR4 expression were observed in different gut segments of diabetic rats. The number of TLR4-IR myenteric neurons, as well as their proportion to pan-neuronal peripherin-IR neurons, was increased in the diabetic duodenum (31.41 ± 2.32 vs. 19.74 ± 1.71; *p* < 0.01), robustly increased in the ileum (63.76 ± 3.17 vs. 28.99 ± 2.44; *p* < 0.0001), and decreased in the colonic ganglia of diabetics (24.15 ± 2.88 vs. 70.05 ± 3.49; *p* < 0.0001) relative to controls (Figure 3 and Figure 4). The insulin treatment was also showing regional effects; while it completely prevented the hyperglycemia-related changes in TLR4 expression in the duodenum (17.97 ± 1.83), and partially in the colon (39.05 ± 4.40), the proportion of TLR4-IR myenteric neurons was even lower in the ileal ganglia of insulin-treated diabetic rats than in that of controls (14.69 ± 1.86 vs. 28.99 ± 2.44; *p* < 0.001) (Figure 4). The number of those ganglia which did not contain TLR4-IR cells strictly followed these changes in diabetic groups. Accordingly, we did not observe TLR4-immunoreactivity in 24.47% of duodenal, only 5.55% of ileal and 18.28% of colonic myenteric ganglia of diabetic rats.

**Figure 5:**
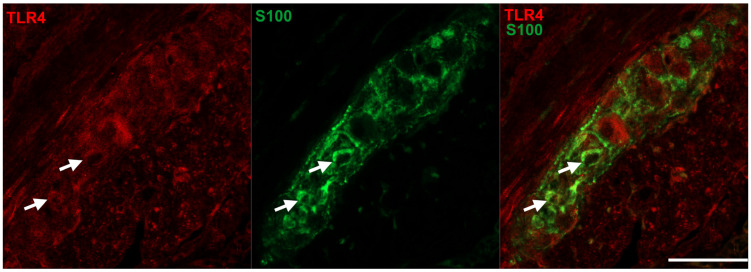
Representative fluorescent micrographs of a paraffin section from diabetic duodenum showing the myenteric plexus after TLR4-S100 double-labelling immunohistochemistry. S100 was applied to label enteric glial cells. Arrows—enteric glia cells, scale bars: 10 μm.

Interestingly, not all of the TLR4-IR cells were also positive for the neuronal marker peripherin, only 95.24% of TLR4-IR cells were also peripherin-IR in the duodenum, 93.04% in the ileum and 97.31% in the colon of controls. Therefore, fluorescent immunohistochemistry using TLR4 and S100 antibodies was performed to elucidate the presence of TLR4 in enteric glial cells. Double-labelling immunohistochemistry on paraffin sections revealed that TLR4 immunoreactivity was clearly visible in myenteric glial cells of the intestinal wall (Figure 5).

### 3.4. Subcellular Localization and Electron Microscopic Quantification of TLR4 Expression in Myenteric Ganglia

**Figure 6:**
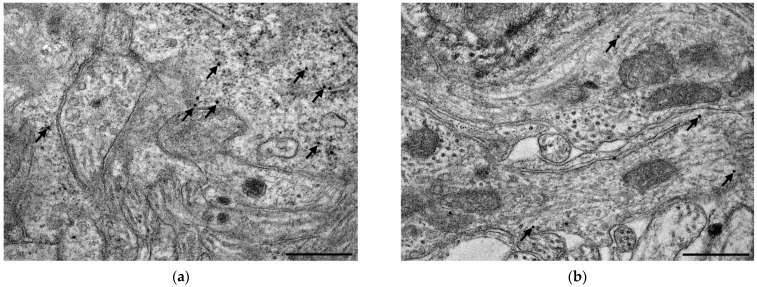

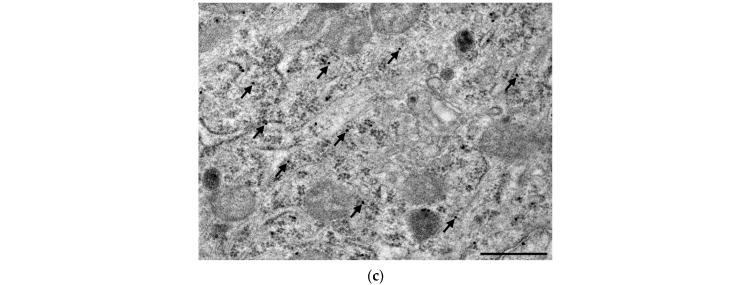
Representative electron micrographs of myenteric neuronal perikaryon and ganglionic neuropil from the ileum of a control rat after TLR4 post-embedding immunohistochemistry. The number of TLR4 labelling gold particles was higher in neuronal perikaryon (**a**,**c**) than in myenteric neuropil (**a**,**b**). Arrows—18 nm gold particles labelling TLR4, scale bars: 500 nm.

The quantitative subcellular evaluation of TLR4 expression was carried out by post-embedding immunogold electron microscopy on ultrathin sections of myenteric ganglia. The 18 nm gold particles labelling TLR4 were often located at the plasma membranes or intracellular membranes in all the investigated gut segments and experimental conditions. However, the subcellular distribution of TLR4-labelling gold particles was not homogenous in the myenteric ganglia, while the number of TLR4 labels was apparently higher in neuronal perikaryon than in myenteric neuropil (Figure 6).

**Figure 7:**
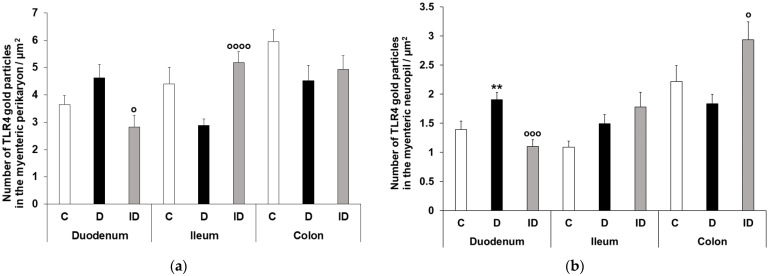
Quantitative evaluation of gold particles labelling TLR4 in perikaryon of myenteric neurons (**a**) and ganglionic neuropil (**b**) from different gut segments of control, diabetic and insulin-treated diabetic rats. TLR4 density increased both in myenteric perykaryon and the neuropil of the diabetic duodenum, decreased in both compartments in the diabetic colon and a subcellular rearrangement of myenteric TLR4-labelling gold particles was revealed in the ileum of diabetics. Statistics: Kruskal-Wallis test with Dunn’s multiple comparisons test. Data are expressed as means ± SEM. ** *p* < 0.01 (relative to controls); ^o^ *p* < 0.05, ^ooo^ *p* < 0.001, ^oooo^ *p* < 0.0001 (between diabetics and insulin-treated diabetics). C—controls, D—diabetics, ID—insulin-treated diabetics.

There were nearly three times more gold particles in the perikaryon than in the neuropil in the duodenum and colon, and four times more in the ileum of controls (Figure 7). In controls, the number of gold particles displayed the same proximal-distal regionality of basic TLR4 expression both in the perikaryon and myenteric neuropil as was seen by fluorescent immunohistochemistry (Figure 7).

A quantitative evaluation of subcellular TLR4 expression confirmed that the TLR4 density increased both in myenteric perikaryon and the neuropil (*p* < 0.01) of the diabetic duodenum, which was completely prevented by insulin treatment in both compartments (Figure 7). Similarly, in the colon of diabetics, a decrease of TLR4 density was observed in both compartments. However, a subcellular rearrangement of myenteric TLR4-labelling gold particles was revealed in the ileum of diabetic animals. Here, TLR4 density displayed a decreasing tendency in the myenteric perikaryon, but an increasing tendency in the neuropil region relative to controls (Figure 7). Insulin had only partially effects in the distal gut segments.

### 3.5. Intestinal Layer-Dependent TLR4 Expression in Tissue Homogenates of Diabetic Rats

**Figure 8:**
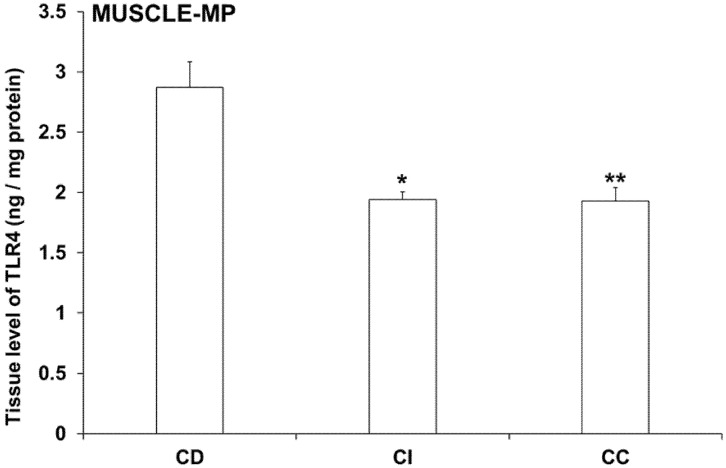
Tissue levels of TLR4 in intestinal smooth muscle layer homogenates including the myenteric plexus from different gut segments of control rats. The TLR4 level was highest in the duodenum, while it was significantly lower in the ileum and colon of controls. Statistics: Kruskal-Wallis test with Dunn’s multiple comparisons test. Data are expressed as means ± SEM. * *p* < 0.05, ** *p* < 0.01 (relative to control duodenum). CD—control duodenum, CI—control ileum, CC—control colon.

In controls, TLR4 tissue levels of MUSCLE-MP homogenates (including the myenteric plexus and intestinal smooth muscle layers) showed a distinct variation from duodenum to colon. TLR4 concentration was 2.87 ± 0.21 ng/mg in the duodenum, while it was significantly lower in the ileum and colon (1.94 ± 0.07 ng/mg, *p* < 0.05; and 1.93 ± 0.11 ng/mg, *p* < 0.01; respectively) (Figure 8).

**Figure 9:**
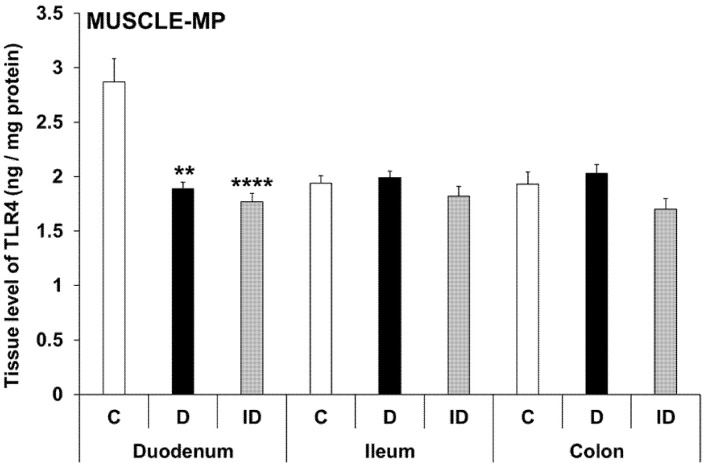
Tissue levels of TLR4 in intestinal smooth muscle layer homogenates including the myenteric plexus from different gut segments of control, diabetic and insulin-treated diabetic rats. In the diabetics, TLR4 concentration was decreased by more than 30% only in the duodenum relative to controls, which was not prevented by insulin. Statistics: Kruskal-Wallis test with Dunn’s multiple comparisons test. Data are expressed as means ± SEM. ** *p* < 0.01, **** *p* < 0.0001 (relative to controls). C—controls, D—diabetics, ID—insulin-treated diabetics.

In diabetic MUSCLE-MP homogenates, TLR4 levels were decreased by more than 30% only in the duodenum relative to controls (1.89 ± 0.06 vs. 2.87 ± 0.21; *p* < 0.01), and this decrease was not prevented by insulin treatment (Figure 8). Meanwhile, the TLR4 concentration did not change in the ileum and colon in either the diabetic or insulin-treated groups (Figure 9).

**Figure 10:**
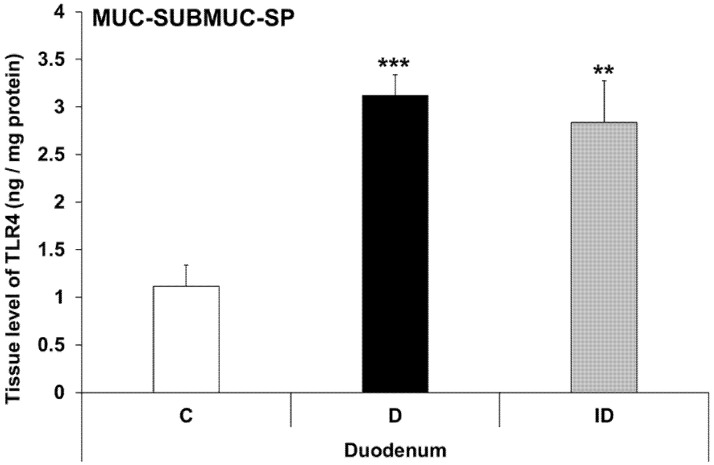
Tissue levels of TLR4 in mucosa and submucosa homogenates, including the submucous plexus, from the duodenum of control, diabetic and insulin-treated diabetic rats. The TLR4 level was robustly increased in diabetic samples of the duodenum relative to controls and was not prevented by insulin replacement. Statistics: Kruskal-Wallis test with Dunn’s multiple comparisons test. Data are expressed as means ± SEM. ** *p* < 0.01, *** *p* < 0.001 (relative to controls). C—controls, D—diabetics, ID—insulin-treated diabetics.

Therefore, TLR4 levels were also measured in other duodenal layers. In MUC-SUBMUC-SP homogenates (including the submucous plexus, mucosa and submucosa layers), an almost triple increase in TLR4 levels was detected in diabetic samples of the duodenum relative to controls (3.12 ± 0.21 vs. 1.11 ± 0.23, *p* < 0.001), which was not prevented by insulin treatment (Figure 10). This section may be divided by subheadings. It should provide a concise and precise description of the experimental results, their interpretation, as well as the experimental conclusions that can be drawn.

## 4. Discussion

STZ, originally derived from *Streptomyces achromogenes* [24], as a diabetogenic agent is suitable to induce hyperglycemia in rodents. Besides other type 1 diabetic animal models [25,26] and despite STZ’s toxic effects not only on pancreatic beta cells but also on other cell types [27,28], it is commonly used to model type 1 diabetes.

Previous studies using the same STZ-induced type 1 diabetic rat model showed that the hyperglycemia-related dysbiosis is gut-region specific [29,30]. This is consistent with our other findings that diabetes damages the enteric nervous system to a different extent along the intestinal tract [6,23,31]. Therefore, in this study we wanted to examine the role of TLR4 in the myenteric plexus and answer the following questions: What proportion of TLR4-positive enteric neurons are present in the different regions of the gut under control conditions and in a chronic hyperglycemic state? Does immediate insulin treatment have any effect on TLR4-expression in the myenteric plexus?

TLRs are produced by a variety of gut cell types, such as epithelial cells, enteroendocrine cells and enteric neurons [32]. Based on literary data, epithelial TLR4 expression is lower in the small intestine but higher in the colon [32,33]. According to our present results, the proportion of TLR4-IR myenteric neurons also showed an increasing tendency along the proximal-distal axis of the intestinal tract in control rats. The reason for the region-specific differences in TLR4 expression is not clear either in the epithelium or in the myenteric ganglia, but it is most likely related to the microbiota of the given intestinal section [13,33,34].

In diabetes, the proportion of TLR4-positive myenteric neurons changed significantly in all observed gut regions. The representation of TLR4-IR neurons was higher in the duodenum and ileum, while it was lower in the colon of diabetics when compared to the controls. It has been shown that the expression of TLR4 is elevated in inflammation [32], and the TLR expression correlates well with changes in microbial composition [33], especially changes within the Gram-negative bacteria. In earlier studies, using the same type 1 diabetic rat model, perturbation of both mucosa-associated and fecal microbiota was demonstrated [29,30]. In a diabetic duodenum, a significant invasion of the genus Mycoplasma was shown in the mucosa-associated microbiota [30], while in the ileum, the composition of luminal microbiota changed significantly by Klebsiella invasion [29]. We propose that the diabetes-related inflammation and dysbiosis may underline changes in TLR4 expression.

This hypothesis is supported by our previous result, in which the expression of TNFα showed the same tendency in myenteric ganglia. TNFα levels were increased in the small intestine and decreased in colonic ganglia in response to diabetes similarly to TLR4 expression [5]. Based on our recent study [35] TNFα causes the diabetes-related effects primarily through TNFR2.

The subcellular distribution of TLR4 in the intestinal epithelium has been studied [36], but has yet to be observed in enteric neurons. In controls, our ultrastructural results showed the same trend as with fluorescent immunohistochemistry: the density of TLR4-labelling immunogold particles was lowest in the duodenum, and highest in the colon, both in myenteric perikaryon and neuropil. When comparing subcellular localizations, the density of TLR4-labelling immunogold particles was higher in the myenteric perikaryon than in the neuropil. We believe that the possible rearrangement of the gold particles between the neuropil and perikaryon draws attention to the influence of TLR4 on enteric neuronal function.

It has been demonstrated in recent years that TLR4 has an effect on neuronal survival in the enteric nervous system [32,37,38]. Vincentini et al. [39] showed that TLR4 activation by LPS administration prevented the antibiotic-induced enteric neuronal loss but did not affect neurogenesis or differentiation. Chronic hyperglycemia itself and its harmful/protective mechanisms influence neuronal survival in myriad ways [6,40,41]. Besides neuronal death, adult neurogenesis also influences the total enteric neuronal number and the ratio of the different subpopulations (e.g., nitrergic, cholinergic) of the enteric neurons [39,42]. Anitha et al. [37] reported that the lack of TLR4 is associated with reduced gastrointestinal motility, and found that these motility problems were caused by damage to the enteric neurons and not the muscle.

According to our results, TLR4 tissue levels of MUSCLE-MP homogenates decreased from duodenum to colon. While these homogenates consist of mainly smooth muscle cells, our data showed that the TLR4 expression was different along the gastrointestinal tract. The diabetic state only affected the duodenum, where the TLR4 tissue levels in the MUSCLE-MP decreased relative to control data. Therefore, the MUC-SUBMUC-SP homogenates were also observed, and our data showed a significant increase of TLR4 levels in the diabetic duodenum compared to controls. This difference in tissue TLR4 concentration among the layers of the intestinal wall may highlight the significance of TLR4 expression in the crosstalk of microbiota, the intestinal epithelium, and the immune, neural, and glial cells in health and disease. This is also proven by knockout studies in which attenuated inflammation was observed in TLR knockout mice [43,44].

More studies are needed to elucidate the role of TLR4 in the different cell types, histological layers, or regions of the gastrointestinal tract, but taken together, these findings highlight the role of TLR4 in normal enteric nervous system function and emphasize its role as a therapeutic target [45,46]. Furthermore, TLR4 not only exerts an influence on the enteric nervous system, but also mediates the effects of gut microbiota and fine-tunes neuro-immune interactions in the whole organism.

## Figures and Tables

**Table 1:** Weight and glycemic characteristics of the experimental groups.

	Body Weight (g)		Blood Glucose Concentration (mmol/L)	
	Initial	Final	Initial	Final (Average)
Controls (n = 15)	235.6 ± 8.47	434.1 ± 9.18 ^a^	4.97 ± 0.31	5.80 ± 0.14
Diabetics (n = 12)	238.2 ± 10.21	353.6 ± 10.94 ^ab^	4.86 ± 0.36	24.73 ± 1.02 ^ab^
Insulin-treated diabetics (n = 12)	231.9 ± 8.79	415.5 ± 8.98 ^ac^	4.83 ± 0.32	11.1 ± 0.99 ^abc^

Statistics: one-way ANOVA with Newman–Keuls test. Data are expressed as mean ± SEM; ^a^ *p* < 0.0001 vs. initial; ^b^ *p* < 0.0001 vs. final controls; ^c^ *p* < 0.0001 vs. final diabetics.

## Data Availability

Dataset available from the corresponding author at bodi.nikolett@bio.u-szeged.hu e-mail address.
